# Effects of Fishing and Regional Species Pool on the Functional Diversity of Fish Communities

**DOI:** 10.1371/journal.pone.0044297

**Published:** 2012-08-31

**Authors:** Gustavo M. Martins, Francisco Arenas, Ana I. Neto, Stuart R. Jenkins

**Affiliations:** 1 Centro Interdisciplinar de Investigação Marinha e Ambiental (CIIMAR), Porto, Portugal; 2 Grupo de Biologia Marina, CIRN & Departamento de Biologia, Universidade dos Açores, Ponta Delgada, Portugal; 3 School of Ocean Sciences, Bangor University, Menai Bridge, Anglesey, United Kingdom; 4 Marine Biological Association, Citadel Hill, Plymouth, United Kingdom; Technical University of Denmark, Denmark

## Abstract

The potential population and community level impacts of fishing have received considerable attention, but little is known about how fishing influences communities’ functional diversity at regional scales. We examined how estimates of functional diversity differed among 25 regions of variable richness and investigated the functional consequences of removing species targeted by commercial fisheries. Our study shows that fishing leads to substantial losses in functional diversity. The magnitude of such loss was, however, reduced in the more speciose regions. Moreover, the removal of commercially targeted species caused a much larger reduction in functional diversity than expected by random species deletions, which was a consequence of the selective nature of fishing for particular species traits. Results suggest that functional redundancy is spatially variable, that richer biotas provide some degree of insurance against the impact of fishing on communities’ functional diversity and that fishing predominantly selects for particular species traits. Understanding how fishing impacts community functional diversity is key to predict its effects for biodiversity as well as ecosystem functioning.

## Introduction

Humans have historically gathered fish and shellfish for subsistence. However, powered by increasing social and economic demands, and more advanced fishery technologies, the impact of fishing has never been as high [1]–[3]. At population levels, it is now clear that fishing directly leads to a reduction in the numbers and size of target species [1], [4], as well as indirectly affecting population dynamics through for example changes in reproductive output [5]. However, the impacts of fishing are not restricted to target populations. Changes made to the population structure of key taxa, often at the top of the food chain and hence distortions of predator-prey ratios, can lead to community-wide alterations via cascading community effects [4]. Notable examples include the regional-scale changes in the structure of rocky shores along the coasts of Chile as result of the exploitation of a key predatory mollusc [1], or profound changes extending down the food-web following the collapse of the Canadian cod stocks [3].

Understanding the role of biodiversity and specifically the impact of species loss on the functioning of ecosystems has been a central topic in the ecological literature over the past two decades. The question of whether the number of species *per se* or the identity of species matters for ecosystem processes, has led to advances in research methodologies [6]–[7] which have allowed scientists to disentangle these two components of diversity. Recent studies have often shown that richness effects (number of species) are generally weak, whilst compositional effects are commonly much stronger [8]–[12]. This suggests that it is the range of species functional traits (functional diversity *sensu* [13]), but not species richness *per se*, that likely underpins a mechanistic link between species diversity and ecosystem functioning. Work in a range of habitats, including experimental grass fields [14] and intertidal macroalgal assemblages [12], [15], provides support for this assertion.

The spatial scale of study is likely to have a significant impact on the relationship between species richness and ecosystem functioning [16]–[17]. At local scales, species sorting processes (e.g. competition) cause a reduction in diversity [18] resulting in a community composed of species whose optimal growth matches the local conditions. The capacity of the community to resist environmental changes is hence reduced [19]–[21]. At larger scales however, the regional species pool provides a source of renewal of local species assemblages that, via immigration and dispersal, can be important for sustaining ecosystem functioning under changing conditions [21]. For example, Naeslund and Norberg [22] showed that communities developed from samples of single origin exhibited important differences in the community structure and in their response to ecosystem processes from communities developed from mixed samples of multiple origins. According to the insurance hypothesis, regionally rich biotas may thus be better adapted at sustaining ecosystem processes against fluctuating environmental conditions compared to depauperate biotas [20], [23].

Here we examine the influence of fishing on functional diversity using an existing dataset [24]–[25] on coastal fish across 25 Atlantic regions. Halpern and Floeter [25] showed that the relationship between regional species richness and richness of functional groups was asymptotic and that several functional groups (different combinations of species traits) either had no or few species (unsaturated), hence species redundancy was limited to relatively few groups. They suggested that understanding differences in species richness among functional groups is particularly important for predicting how human activities affect biological communities and ecosystem functioning. We re-examine this dataset using a continuous measure of functional diversity (FD *sensu* Petchey and Gaston [26]). We then examine the effect of fishing on functional diversity by deliberately deleting commercially targeted species from the regional species pool and determining the impact of this process on functional diversity. We test the hypothesis that regional species richness functions as insurance to species loss and compare the effects of fishing to that of equivalent but random species deletions. We further examine what functional traits are preferentially removed by fishing in order to ascribe the potential impact of fishing on ecosystem functioning.

## Results

### Regional Species Richness and Functional Diversity Relationship

**Figure 1:**
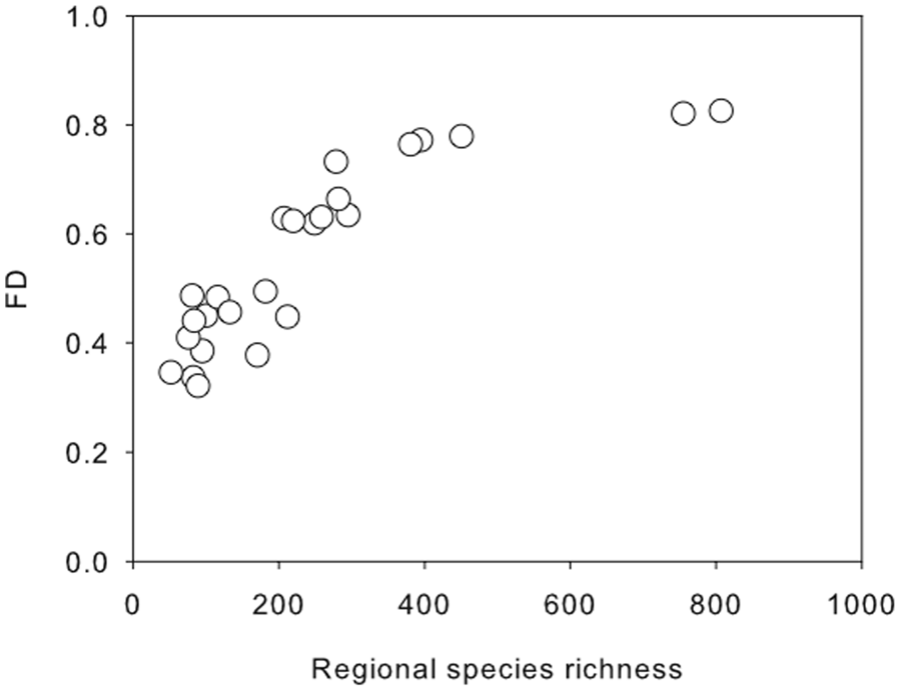
Relationship between functional diversity (FD) and regional species richness. Each point represents one the 25 communities examined.

The relationship between regional species richness and functional diversity (FD *sensu* Petchey and Gaston [26]) was asymptotic (Fig. 1). FD generally increased with regional species richness but this effect was more pronounced at lower levels of richness and beyond a regional richness of 400 there was little apparent increase in FD.

### Effect of Species Loss and Regional Species Richness on Functional Diversity

**Figure 2:**
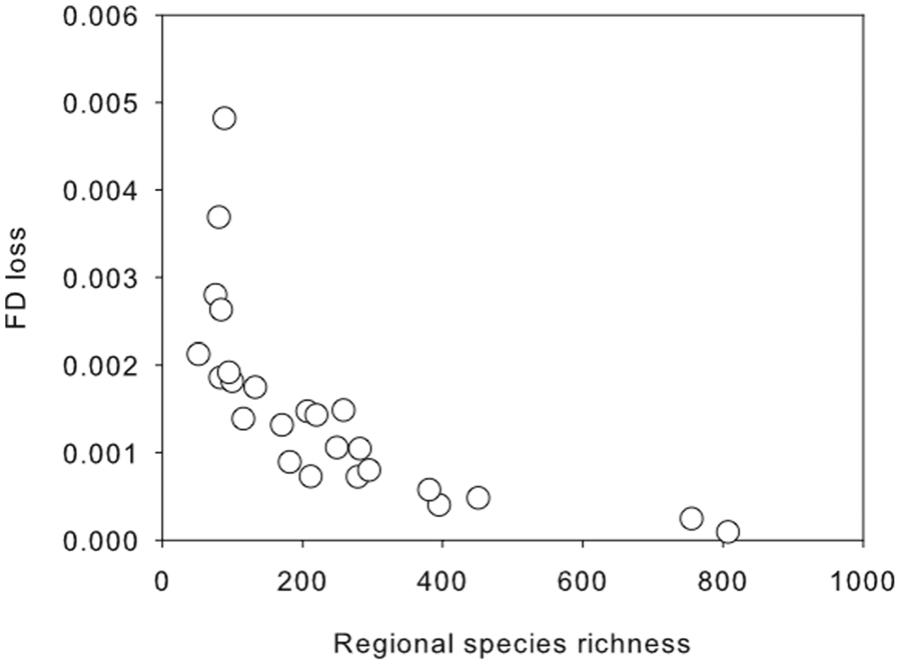
Loss of functional diversity (FD) and regional species richness. Loss of FD is the difference in FD before and after the removal of 1 random species. For each region, the value of FD was calculated as the mean of 100 iterations. See methods for further details.

On average, the removal of a single species at random led to a decrease of 0.0015±0.0002 (mean ± SE) in FD. Loss of FD, however, differed with regional species richness (Fig. 2). Loss of FD as a consequence of removing a single species was substantially reduced in speciose as compared to depauperate regions supporting the hypothesis that regional species richness may provide insurance against species loss for ecosystem processes.

### Effect of Fishing and Regional Species Richness on Functional Diversity

**Figure 3:**
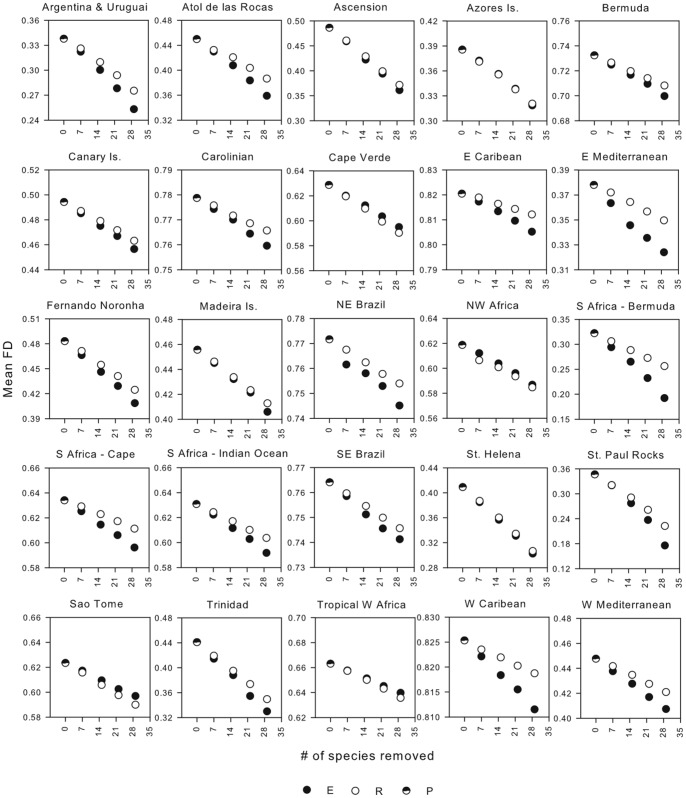
Relationship between functional diversity (FD) and fishing pressure. Each point represents the mean FD (n  = 100 iterations) of communities after removing 7, 15, 22, and 29 species. Species removals were done by either randomly drawing commercially exploited species only (E – filled circles) or at random from the entire species pool (R – open circles). FD of pristine communities (no species removals) is also shown (P – half-filled circles). All regressions were significant at α  = 0.05.

For all the communities examined, removal of species led to significant decreases in FD, (all regressions *P*<0.05) (Fig. 3). This was true when only species targeted by commercial fishing were selected for removals but also when species removals were drawn randomly from the entire species pool. The rate at which FD was lost, however, differed according to the way species were lost (exploited versus random). In 19 out of the 25 communities examined, the removal of exploited species caused a significantly faster/greater reduction (greater slope or lower intercept, respectively) in FD as compared to random species removals (Table 1). Only in 4 out of the 25 communities was the effect of removing exploited species lower than that of random species removals (Table 1). Hence, the selection of exploited species for removal generally had a greater negative effect on FD than expected by random species losses.

**Table 1:** Comparison of the effects of random versus exploited species removals on communities FD.

Community	Richness (covariate)	E vs. R (intercept)	Slope	Outcome
Argentina and Uruguay	1045.20**	86.33***	24.30***	Slope R < E
Atol de las Rocas	598.55**	77.36***	26.66**	Slope R < E
Ascension	7009.90**	49.19***	12.22*	Slope R < E
Azores Islands	742.34**	0.032	0.57	No difference
Bermuda	914.80**	68.90**	21.95*	Slope R < E
Canary Islands	1718.00***	82.28***	13.28*	Slope R < E
Carolinian	770.26**	99.15**	30.07**	Slope R < E
Cape verde	1933.60**	43.85**	11.19*	Slope E < R
Eastern Caribean	1301.10**	437.54***	101.19***	Slope R < E
Eastern Mediterranean	567.29**	374.55***	40.41**	Slope R < E
Fernando Noronha	2179.30***	156.39***	25.04**	Slope R < E
Madeira Islands	969.58**	10.93*	5.27	R > E
NE Brazil	148.79**	43.43***	1.15	R > E
NW Africa	510.67**	22.02*	1.44	E > R
South Africa – Benguela	399.27**	154.14***	47.26**	Slope R < E
South Africa – Cape	5225.50***	1633.60***	294.11***	Slope R < E
South Africa – Indian Ocean	2169.50**	272.21***	75.94***	Slope R < E
SE Brazil	881.73**	71.64**	9.64*	Slope R < E
St. Helena	2950.20**	8.47	0.39	No difference
St. Paul Rocks	300.89**	17.24*	10.72*	Slope R < E
São Tomé	2516.90***	156.24***	33.08***	Slope E < R
Trinidad	1155.30**	55.35*	13.35*	Slope R < E
Tropical West Africa	4181.90***	71.71***	34.71**	Slope E < R
Western Caribean	955.57***	532.47***	130.84**	Slope R < E
Western Mediterranean	3350.30***	714.92***	117.45***	Slope R < E

One-way ANCOVA comparing the slopes and intercepts of regressions (between FD and community richness after the removal of 7, 15, 22 and 29 species) when species were removed by selecting only exploited versus randomly (E vs. R) from the regional species pool. All terms tested against the residual. Table shows only *F*-values and significance values as * *P*<0.05, ** *P*<0.01, and *** *P*<0.001.

**Figure 4:**
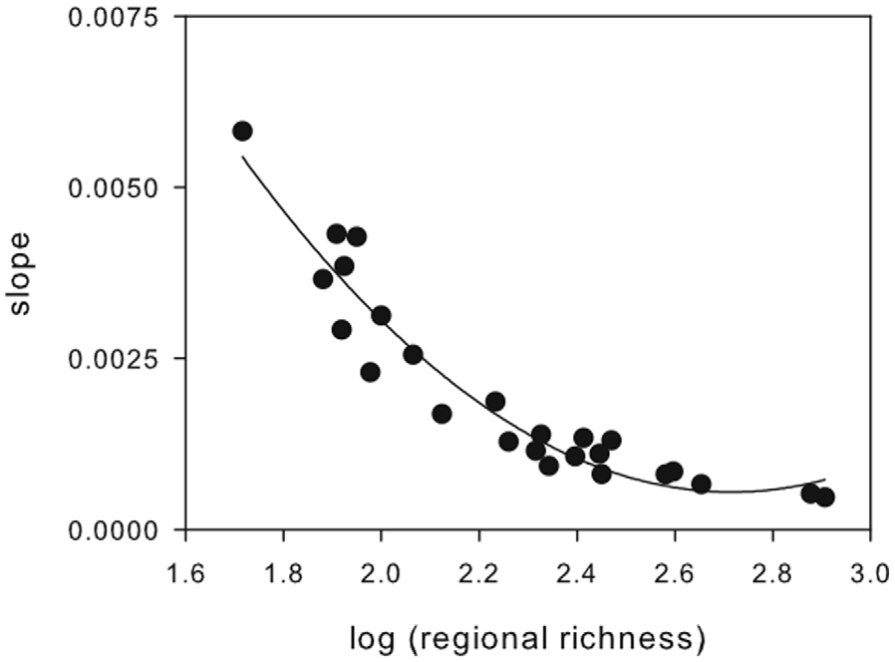
Rate of FD loss and regional species richness. Rate of FD loss corresponds to the slope of the relationship between FD and community richness after removal of 7, 15, 22 and 29 exploited species. A greater slope thus indicates that FD is lost faster as the number of species removed increases.

**Figure 5:**
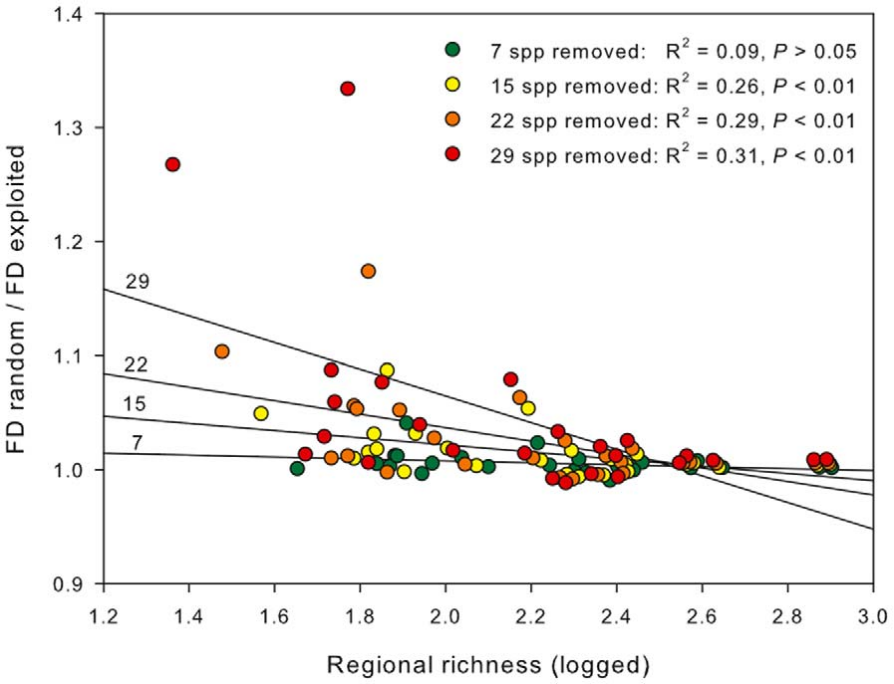
Magnitude of FD loss, regional species richness and fishing intensity. Variation in the magnitude of FD loss between random versus exploited species removals with regional species richness for each level of fishing intensity (number of species removals).

At a larger-scale, there was a significant quadratic relationship between the rate of FD loss (slope of the relationship between FD and number of exploited species removed) and regional species richness (Fig. 4, *F*_2,22_ = 130.18, *P*<0.001, *R* ^2^ = 0.92) indicating that loss of FD as a consequence of fishing is faster in species poor regions. It was also evident that the magnitude of such an effect in relation to random species removals (FD random/FD exploited) was accentuated in species poor regions when pressure on ecosystem (fishing intensity) increased (Fig. 5, Table 2).

**Table 2:** Comparison of the magnitude of FD loss and regional species richness.

Source	Df	MS	*F*	*P*
Regional richness (RR)	1	4.89×10^−2^	30.01	<0.01
Level of removal (LR)	3	5.57×10^−3^	3.42	<0.05
RR × LR	3	7.15×10^−3^	4.39	<0.05
Residual	92	1.63×10^−3^		

One-way ANCOVA comparing the magnitude of the ratio between FD random and FD exploited for different levels of species removals (7, 15, 22 and 29 species removals) as a function of regional species richness (logged). All terms tested against the residual.

### Trait Comparison between Commercially Targeted and Non-targeted Species

When considering the species traits examined (Table 3), commercially exploited species had a significantly greater maximum body size than non-exploited species and were dominated by deep-water species whereas shallow-water species were underrepresented among the exploited species list. In addition, there was proportionally a greater number of macroalgal browsers, macrocarnivores, omnivores, strict piscivores and sand invertivores in targeted species. In contrast, the number of mobile benthic invertivores, nocturnal planktivores and territorial herbivores was proportionally lower among the exploited species. For the remainder of the species functional traits, the proportional number of exploited and non-exploited species was similar (Table 3). Thus, by selectively targeting particular species functional traits, fishing is causing a larger loss of FD than would be expected if species were selected at random.

**Table 3:** Numbers of exploited and non-exploited fish species for each trait.

Category	Trait	Non-exploited	Exploited	Test	Proportion of species
Size	Maximum size	17.0 (21.3)	62.3 (46.0)	No overlap*	E > NE
Depth	Very shallow	234	37	40.38*	E < NE
	Shallow	259	44	40.58*	E < NE
	Mid	202	118	3.10	E = NE
	Deep	207	152	12.20*	E > NE
	Very deep	250	209	26.02*	E > NE
Diet	Diurnal planktivores	66	24	1.43	E = NE
	Excavators	1	3	3.24	E = NE
	Macroalgal browsers	1	8	12.78*	E > NE
	Macro carnivores	50	124	101.57*	E > NE
	Mobile benthic invertivores	749	139	86.55*	E < NE
	Nocturnal planktivores	29	0	13.98*	E < NE
	General omnivores	74	87	29.60*	E > NE
	Strict piscivores	15	60	70.92*	E > NE
	Sand invertivores	65	73	23.12*	E > NE
	Scraper herbivores	22	18	2.67	E = NE
	Coral/colonial sessile invertivores	44	16	0.97	E = NE
	Spongivores	3	5	3.20	E = NE
	Territorial herbivores	24	1	9.29*	E < NE
	Turf grazers	9	2	1.05	E = NE

Comparison of the numbers of exploited (E) and non-exploited (NE) species for each selected trait. The χ^2^ test-of-independence was used for all tests but that of size, which used the overlap of the 95% confidence intervals. The size of the exploited and non-exploited species groups was 560 and 1152 respectively. For size, values indicate mean (± s.d.).

* significant test (α  = 0.05). See Halpern and Floeter [25] supplementary online material for further details on traits.

## Discussion

Results generally suggested two main outcomes: (i) loss of functional diversity due to the removal of targeted species is, to some extent, reduced in the more speciose regional biota and, (ii) the selective removal of commercially exploited species leads to a significantly greater reduction in functional diversity than equivalent but random species removals.

Halpern and Floeter [25] showed that many functional groups either were vacant (no species) or had few species, and that species redundancy was hence restricted to few functional groups. Moreover, when considering the relationship between regional species richness and community functional diversity, most communities fell in the linear part of the graph (see Fig. 1) suggesting that in most regions functional diversity will decrease as species are lost. Hence it comes at no surprise that the removal of targeted species led to a reduction in communities’ functional diversity and that this was accentuated in the species poor regions. This supports the hypothesis that diversity of regional biotas functions as a buffer to ecosystem disruption under changing environmental conditions [20], [23]. The above is supported by the work of Worm et al. [27] who showed that stock collapse was greater, and recovery was slower, in regions with naturally depauperate biotas. In contrast, Mora et al. [28] have recently showed that for a given level of human pressure, standing stock of reef fish (a surrogate for ecosystem functioning) decreased with local diversity.

Two fundamental differences could explain the discrepancy in results; While the study of Mora et al. [28] focused on local richness, that of Worm et al. [27], and our study have focused on regional richness and it is still unclear how results from smaller spatial scales can be scaled-up to larger scales [16]. In addition, the work of Mora et al. [28] used human density as a means to infer human pressure. This, as shown by these authors, was not only related to the levels of fishing, but also with the amounts of fertilizers consumed and urbanisation. In contrast, our study and that of Worm et al. [27] have focused on the effects of fishing alone. While fishing has been shown to be highly selective, predominantly targeting specific functional groups, it is not clear how agriculture (e.g. via nutrient run-off) and coastal urbanisation affect the structure of fish assemblages.

Although we did not assess the effects of diversity on ecosystem functioning, our results provide stronger evidence for the importance of species richness than many small-scale experimental studies in which the effects of richness are often relatively weak [8]–[12]. Most small-scale experimental studies have used synthetically assembled communities and it is not clear as to how results from such small-scale manipulative studies may be scaled-up to natural communities. Moreover, empirical studies have been biased towards the study of species at lower trophic levels (e.g. plants and algae) despite the fact that strong interactions may be more common among large animals at higher trophic levels [29]. Vulnerability to extinction is also predicted to increase with both body size and trophic level [4], [30]–[33]. Since richness of large predators is naturally low, a few extinctions may thus result in the loss of the entire top predator trophic level, with disproportionately large effects on ecosystem properties and processes [31], [34]. This view is supported by work done by Petchey et al. [35] with the British avian assemblages, which generally showed no functional redundancy. Halpern & Floeter [25] also noted important differences in species richness among functional groups of reef fish communities and suggested these could be particularly important for understanding the effects of human activities on biological communities and ecosystem functioning as is here shown.

Processes affecting the regional species biota have been investigated since seminal work by MacArthur and Wilson [36] and it is now generally accepted that latitude, habitat isolation and area all affect patterns of species distribution [36]–[39] and that dispersal rates have a key role in influencing the extent of spatial insurance in diversity across heterogeneous landscapes [20]. From these, it may be predicted that susceptibility of reef fish communities to ecosystem disruption as a result of overfishing may be greater in isolated islands and higher-latitude regions as compared to continental and lower-latitudinal regions. Moreover, some species traits also correlate with geography. For instance, species body size tends to positively correlate with latitude and isolation; this has been shown for both birds [40] and fish [41]–[42]. Since fishing is generally non-random and tends to target larger-bodied species [4], it may be expected that isolated and higher latitude biotas will suffer the greatest from fishing. In fact, Fisher et al. [41]–[42] provide empirical evidence for a greater impact of fishing on species body size at higher latitudes, which has even resulted in the disruption of natural latitudinal gradients in fish body size.

The effects of fishing on functional diversity were surprisingly striking when viewed in comparison to the effects of random species removals. These results suggest that if fishing removed species from all functional groups equally (i.e. random species loss) its impact would have been much reduced. Numerous studies have in the past shown that large predatory fish contribute to the bulk of the catches [4]. Our study corroborates such findings and adds to the wider literature by showing that a proportionally greater number of deep-water species tended to be targeted by fisheries (probably because many shallow water species have no commercial interest) whilst also revealing that species sharing some functional traits (e.g. small mobile benthic invertivores) were proportionally less targeted by commercial fishing. This indicates that such groups of species (e.g. small invertivores) do not constitute the bulk of commercial catches, and this may have unexpected ecosystem-level impacts because it will strengthen the distortion of species ratios (e.g. predator-prey ratio). The above suggests that fishing has an exacerbated impact on functional diversity due to its selective nature concerning the selected species traits. It is unclear if our results can be extrapolated to other fish communities (e.g. freshwater, deep-sea fish communities), but results will likely depend on the numbers of species, the diversity of ecological traits and on the selective nature of fishing for a given community.

Our approach of removing species from regional species list as a proxy for fishing effects appears rather dramatic. However, even though extinctions in the sea may be rare [43]–[45], impairment of biological functions does not require extinctions to occur. Accumulating evidence has shown that significant reductions in the numbers and size of exploited species as a result of overfishing can impair species role on ecosystem functioning [46] leading to noticeable and long-lasting impacts on marine ecosystems [1], [3]. Fishing has also been shown to affect important ecosystem properties such as resilience [47]–[48]. For instance, empirical evidence from tropical coral reefs shows that the reduction in size of parrotfish species as a consequence of fishing has a detrimental effect on their ability to control macroalgae [46], in turn affecting the resilience of coral reef systems [49]. Given the perceived importance of fish for community processes it is noteworthy that the effects of species removals on communities’ functional diversity were remarkably clear even when only a few species were removed.

## Materials and Methods

### Data Acquisition

Species lists for a total of 25 regions haphazardly distributed across the Atlantic were extracted from Floeter et al. [24]. According to these authors, these data provide the most comprehensive species list for Atlantic reef communities and include cryptic, rare and endemic species as well as common and abundant ones [24]. This or part of this dataset has been previously used in biogeographical [24], [50]) and ecological [25] studies. For further details regarding this dataset refer to the above-mentioned literature.

Species were classified according to standard trophic groups (14 groups) and maximum depth (5 groups) as in Halpern and Floeter [25] but we replaced their categorical classification of species maximum body size by the species-specific maximum body size. Unlike them, we used a continuous measure of functional diversity, which avoids the classification of species into closed functional groups and makes a better use of continuous variables (e.g. maximum body size) in distinguishing among similar species [26], [51]. Calculation of functional diversity is not affected by the simultaneous use of categorical and continuous variables since the former are transformed into binary variables *a priori*.

The species traits used in determining functional diversity are related to the acquisition and utilisation of resources: trophic groups directly differentiate among groups of species based on their diet and where and how they acquire their food (e.g. benthos vs. water column); fish body size determines energetic requirements and is an important predictor of ecosystem processes [41]; depth is directly related to where species acquire resources. Fish body size and depth help distinguish among species classified within the same trophic group and are respectively related to species α- and β-niches (*sensu* Ingram and Shurin [52]).

Species were identified as exploited or non-exploited by commercial fisheries. Such information and species specific maximum body-size was obtained using FishBase 2000 dataset [53] and published literature.

### Measuring Functional Diversity

For each region, functional diversity was calculated following the procedures described by Petchey and Gaston [26], [51]. Calculations were done using the software R and the code provided in the authors’ webpage. In short, the species by trait matrix was converted into a distance matrix to produce a dendrogram that depicts the functional relationships among fish assemblages. Functional diversity (FD *sensu* Petchey and Gaston [26]) is the total branch length needed to join all species in an assemblage and is standardised to range between 0 (assemblages composed of 1 species) and 1 [26], [51].

FD aims to quantify resource use complementary and has been suggested by Petchey et al. [14] to generally perform better as a predictor of ecosystem functioning than alternative methods. However, since methodological choices may affect estimates of functional diversity [54], we tested different clustering and resemblance methods as well as alternative measures of functional diversity. However results were robust to methodological choices (see File S1 and Table S1). For clarity, we present only results as produced by FD based on the Euclidean distance and unweighted pair-group method with arithmetic mean. For all analyses, the trait matrix was standardized (mean  = 0, variance  = 1) so that all traits were similarly weighted.

### Data Analysis

For all analyses, calculation of FD after species removals was done by creating 100 random communities from the pool of species available (either the exploited or the entire species list) for each region, while controlling for species richness. Visual inspection of standard deviations confirmed that the 100 iterations were sufficient to guarantee simulation stability. The mean FD calculated from the 100 iterations was then used in the analyses.

To test the hypothesis that speciose regions are less susceptible to species loss, we estimated the FD of the community after removing one species at random (100 times for each region). The difference in FD before and after the removal of one species (mean FD loss) was then regressed against the regional species richness.

To test the hypothesis that fishing reduces communities’ functional diversity we estimated mean FD for each region after the deletion of 7, 14, 22 and 29 species. These figures correspond to approximately 25, 50, 75 and 100% of the commercially exploited species in the most species-poor region. The maximum level of species removals (29 species removed in each region) corresponded, on average, to an overall reduction of 19.8% ±2.7 (mean ± SE, n  = 25) of the regional species richness across all regions. Removal of species was done in two distinct ways: either by limiting the removal of species to the list of commercially exploited species for each region, or by randomly removing species from the entire species pool (including both exploited and non-exploited species). This was important to distinguish the effects of fishing from those of pure species loss.

Standard regression analyses were used to examine the relationship between mean FD and fishing intensity (7, 15, 22 and 29 species removed) for each region. Analysis of covariance (ANCOVA) was used to test for differences in slope and intercept between the two distinct ways via which species were removed (exploited vs. random). Slopes of the relationship between mean FD and species loss were then regressed (polynomial regression) against regional species richness (logged-transformed to meet the assumption of linearity [55]) to test the hypothesis that the rate of FD loss (slope) varied with regional species richness the prediction being that FD loss is faster in species-poor regions than in speciose ones. We further used ANCOVA to test if the magnitude of the difference in FD loss between the selective (exploited) and random species removals (mean FD random/mean FD exploited) differed among fishing levels (numbers of species removed) across the gradient of regional species richness.

To examine the effects of fishing on the distribution of species functional traits the χ^2^-test of independence was used to compare the proportional number of exploited and non-exploited species for each individual categorical trait. For maximum size (a continuous trait), this comparison was done using the mean and the overlap of the 95% confidence intervals.

## Supporting Information

File S1 Robustness of results – Description of the comparison of the results as estimated by different measures of functional diversity.
(DOCX)

Table S1 Correlation coefficients between estimates of functional diversity as estimated by FD, FAD and PS.
(DOCX)
