# Supplementary material for: Effects of Fishing and Regional Species Pool on the Functional Diversity of Fish Communities
Source: PLoS One. 2012 Aug 31;7(8):e44297. doi: 10.1371/journal.pone.0044297 (PMC3432072; doi:10.1371/journal.pone.0044297)
Supplement: File S1 — Robustness of results – Description of the comparison of the results as estimated by different measures of functional diversity. (DOCX) [file pone.0044297.s001.docx]

**SUPPORTING INFORMATION**

**File S1. Robustness of results – Comparison of the results as estimated by different measures of functional diversity**

It has been suggested that methodological choices may affect estimates of functional diversity [1]. To test how robust results were to methodological choices we compared 3 clustering methods (unweighted pair-group method with arithmetic mean (UPGMA), single linkage and complete linkage) and two different resemblance measures (Euclidean and Gower). The combination of Euclidean distance and UPGMA clustering produced a dendrogram with the highest cophenetic correlation (0.98) [2] and was hence used throughout.

In addition, analyses were redone using two multi-trait alternative measures of functional diversity: FAD [3-4] and PS [5]. Both FAD and PS were calculated using the software FDiversity [6]. As shown in Table S1, estimates produced by the three methods (FD, FAD and PS) were highly correlated indicating high collinearity. This suggests that results were robust to variations in methodological choices.

REFERENCES

1 Poos MS, Walker SC, Jackson DA (2009) Functional-diversity indices can be driven by methodological choices and species richness. Ecology 90: 341-347.

2 Petchey OL, Gaston KJ (2006) Functional diversity: back to basics and looking forward. Ecol Lett 9: 741-758.

3 Walker BH, Kinzig A, Langridge JL (1999) Plant attribute diversity, resilience, and ecosystem function: the nature and significance of dominant and minor species. Ecosystems 2: 95-113.

4 Walker BH, Langridge JL (2002) Measuring functional diversity in plant communities with mixed life forms: A problem of hard and soft attributes. Ecosystems 5: 529-538.

5 Podani J, Schmera D (2006) On dendrogram-based measures of functional diversity. Oikos 115: 179-185.

6 Casanoves F, Pla L, Di Rienzo JA, Diaz S (2011) FDiversity: a software package for the integrated analysis of functional diversity. Methods Ecol Evol 2: 233-237.

7 Petchey OL, Gaston KJ (2002) Functional diversity (FD), species richness and community composition. Ecol Lett 5: 402-411.
