# Supplementary material for: Effects of Fishing and Regional Species Pool on the Functional Diversity of Fish Communities
Source: PLoS One. 2012 Aug 31;7(8):e44297. doi: 10.1371/journal.pone.0044297 (PMC3432072; doi:10.1371/journal.pone.0044297)
Supplement: Table S1 — Correlation coefficients between estimates of functional diversity as estimated by FD, FAD and PS. (DOCX) [file pone.0044297.s002.docx]

**SUPPORTING INFORMATION**

**Table S1. Correlation coefficients between estimates of functional diversity as estimated by FD, FAD and PS.**

|  | **FD** | **FAD** | **PS** |
| --- | --- | --- | --- |
| FD | 1 |  |  |
| FAD | 0.84 | 1 |  |
| PS | 0.99 | 0.85 | 1 |
